# L-carnitine modulates molecular responses associated with inflammation, endoplasmic reticulum stress, and neurotrophic signaling in cerebral cortex of neonatal *Gcdh*^−/−^ mice exposed to glutaric and quinolinic acids

**DOI:** 10.1007/s11011-026-01973-y

**Published:** 2026-09-05

**Authors:** Ediandra Tissot Castro, Ângela Zanatta, Andrey Vinicios Soares Carvalho, Amanda Uggeri de Moraes, Carlos Alexandre Netto, Guilhian Leipnitz, Alexandre Umpierrez Amaral, Carmen Regla Vargas, Rafael Teixeira Ribeiro, Moacir Wajner

**Affiliations:** 1 PPG Ciências Biológicas: Bioquímica, Departamento de Bioquímica, Instituto de Ciências Básicas da Saúde, Universidade Federal de Rio Grande Do Sul, Rua Ramiro Barcelos, 2600, Prédio 21111, Porto Alegre, RS 90035-003 Brazil; 2 Serviço de Genética Médica, Hospital de Clínicas de Porto Alegre, Porto Alegre, Brazil; 3 PPG Atenção Integral À Saúde (UNICRUZ/URI-Erechim/UNIJUÍ), URI, Erechim, Brazil; 4 PPG Ciências Farmacêuticas, UFRGS, Porto Alegre, Brazil

**Keywords:** Glutaric acid, *Gcdh*^*−/−*^ mice, Cerebral cortex, Quinolinic acid, Inflammatory response, Neurotrophins

## Abstract

Glutaric acidemia type 1 (GA I) is caused by deficient activity of glutaryl-CoA dehydrogenase, leading to predominant accumulation of glutaric acid (GA) in the brain. GA I patients present progressive neurological deterioration whose pathophysiology is only partially elucidated. We investigated whether intracerebral GA administration, alone or combined with quinolinic acid (QA), a pro-inflammatory intermediate of the kynurenine pathway, could alter the expression of genes associated with inflammatory signaling, endoplasmic reticulum (ER) stress, and neurotrophic support in cerebral cortex of wild-type (WT) and *Gcdh*^*−/−*^ mice. We also tested the effects of L-carnitine (Carn) on these parameters. GA alone increased mRNA levels of the genes encoding NF-κB (*NFKB1*), COX-2 (*PTGS2*), iNOS (*NOS2*), and TLR2 (*TLR2*) in both genotypes, while reducing those of IL-10 (*IL10*) and VEGF-A (*VEGFA*) only in Gcdh^−/−^ mice. Combined QA + GA treatment produced a larger response, including increased TNF-α (*TNF*), IL-1β (*IL1B*), IL-6 (*IL6*)*,* NLRP3 (*NLRP3*), CHOP (*DDIT3*) and PERK (*EIF2AK3*) mRNA levels in the Gcdh^−/−^ mice, besides reducing IκBα (*NFKBIA*), IL-10 (*IL10*) and BDNF (*BDNF*) expression in both genotypes, and VEGF-A (*VEGFA*) in the *Gcdh*^*−/−*^ mice. We also found that lysine (Lys) and QA treatment elevated TNF-α, IL-1β, and IL-6 protein levels in *Gcdh*^*−/−*^ mice. Carn prevented or attenuated most transcriptional alterations induced by QA + GA and reduced cytokine protein elevations elicited by Lys + QA administration. These findings indicate that Carn modulates acute molecular responses related to inflammation, ER stress, and neurotrophic factors in the cerebral cortex of the GAI mouse model.

**Supplementary Information:**

The online version contains supplementary material available at https://doi.org/10.1007/s11011-026-01973-y.

## Introduction

Glutaric acidemia type I (GA I, OMIM #231,670) is a severe inherited neurometabolic disorder caused by deficiency of glutaryl-CoA dehydrogenase (GCDH, EC 1.3.99.7) activity, leading to predominant accumulation of glutaric (GA) in the brain and biological fluids and secondary to carnitine deficiency (Scriver et al. 2001; Boy et al. 2023). Its worldwide incidence is 30,000–100,000 children, which makes it one of the more common inherited metabolic disorders (Lindner et al. 2006; Strauss et al. 2020).

Untreated infantile-onset GA I patients usually present with acute encephalopathic crises mostly triggered by intercurrent infectious illness, fasting, or other physiological stressors associated with inflammatory processes. Acute striatal deterioration occurs most commonly between ages three months and three years and are usually triggered by a febrile illness, such as infectious, associated with catabolism (Kölker et al. 2006). Neurological deterioration accompanied by insidious striatum and cortical abnormalities without a well-defined triggering event also occur in these patients (Boy et al. 2019). Late-onset GA I (symptoms only occur after six years of life) manifest with nonspecific neurologic signs associated with progressive brain abnormalities beginning at the age of six years (Boy et al. 2019). These chronic presentations characterized by insidious progressive striatum and cortical abnormalities and atrophy are associated with demyelination of the central nervous system (Neumaier-Probst et al. 2004; Funk 2005; Strauss et al. 2007; Harting et al. 2009; Garbade et al. 2014).

Long-term therapy is based on low-lysine diet and carnitine supplementation to accelerate the detoxification of the glutaryl-CoA derivatives GA and 3-hydroxyglutaric acid (3-OHGA), whereas aggressive emergency treatment during metabolic crises aims to rapidly avert catabolism and minimize CNS exposure to the toxicity of the accumulating organic acids (Boy et al. 2013, 2023).

Although yet poorly known, the pathogenesis of the brain damage in GA I seems to be related to the high brain levels of the major organic acids accumulating in GA I, namely GA and 3-OHGA. In this particular it has been shown that these endogenous metabolites disturb redox and energy homeostasis besides inducing excitotoxicity in rat brain (Porciúncula et al. 2004; Magni et al. 2009; Olivera-Bravo et al. 2011; Jafari et al. 2011; Wajner 2019; Amaral et al. 2019; Wajner et al. 2019; Seminotti et al. 2020, 2022; Castro et al. 2024).

A genetic knockout mice model of GA I was developed with complete loss of GCDH activity (*Gcdh*^*−/−*^) (Koeller et al. 2002), and later improved by a high protein or Lys chow resulting in higher brain GA and 3-OHGA concentrations that provoked neuronal loss, myelin disruption (spongiform leukoencephalopathy), and gliosis, mostly in the deep cortex and striatum (Zinnanti et al. 2006, 2007). Disruption of energy and redox homeostasis, apart from altered glutamatergic and GABAergic systems have been shown in the brain of *Gcdh*^*−/−*^ mice exposed to a short-term or long-term Lys overload (Kölker et al. 2002; Seminotti et al. 2012, 2013, 2014; Amaral et al. 2012a, b, 2015; Lagranha et al. 2014; Busanello et al. 2014; Rodrigues et al. 2015; Vendramin Pasquetti et al. 2017). Behavioral alterations have been also found in adult *Gcdh*^*−/−*^ animals reflecting a permanent brain damage (Busanello et al. 2013). Furthermore, *Gcdh*^*−/−*^ mice receiving an acute intracerebral injection of GA on postnatal day 1 presented disturbed neuromotor delay, reflected in abnormal gait, response to negative geotaxis, cliff aversion, righting reflex, and muscle tone, as well as long-standing short- and long-term memory impairment (Castro et al. 2024).

Although the severity of the clinical phenotype of GA I patients depends on the frequency of encephalopathic crises during infancy and childhood, which are mostly triggered by infectious/inflammatory processes and lead to striatum necrosis, practically nothing is known on the impact of these acute inflammatory processes on the cortical alterations observed in GA I. Noteworthy, free carnitine depletion are also observed in these patients as a consequence of the combination of carnitine with glutaric acid producing glutarylcarnitine, and is thought to contribute to hypotonia and other symptoms (Christensen et al. 2004; Strauss et al. 2020; Märtner et al. 2021).

Furthermore, it is stressed that during episodes of metabolic decompensation triggered by infections or immunization the kynurenine pathway (KP) is activated by inflammatory cytokines, leading to overproduction of kynurenic acid, kynurenine, and particularly quinolinic acid (QA). When in excess, these compounds may be toxic to the central nervous system through different pathomechanisms (Lugo-Huitrón et al. 2013), such as activation of N-methyl-D-aspartate receptors (NMDAr), which secondarily leads to excessive free radical production and bioenergetics dysfunction (Ribeiro et al. 2006; La Cruz et al. 2012). In what concerns to GA I, it was previously postulated that QA is involved in the neuropathology of this disease (Varadkar and Surtees 2004), but so far, very little has been explored in this particular. However, it was previously reported that intracerebral injection of QA elicits an inflammatory response and induces oxidative stress and bioenergetics dysfunction in brain of young adult *Gcdh*^*−/−*^ mice (Seminotti et al. 2016; Amaral et al. 2018).

Noteworthy, recent studies using human GCDH-deficient neuronal cells and gene therapy-based animal models have expanded the understanding of GA I pathophysiology and reinforced the contribution of the accumulated metabolites to cellular and neurological injury (Mateu-Bosch et al. 2024; Segur-Bailach et al. 2025; Saad et al. 2026). In parallel, advances in kynurenine pathway research have highlighted QA as an important mediator linking immune activation to excitotoxicity, oxidative stress, and neuroinflammation (Pocivavsek et al. 2024). Previous studies on GA I pathogenesis have predominantly examined the separate effects of accumulated GA or QA (Seminotti et al. 2016; Amaral et al. 2018; Castro et al. 2024). However, to the best of our knowledge, no previous in vivo study has comprehensively characterized the molecular responses to GA alone or in combination with QA in the cerebral cortex of neonatal Gcdh^−/−^ mice. Therefore, we examined the effects of these acute treatments on neuroinflammatory signaling, endoplasmic reticulum stress-related pathways, and neurotrophic factors. Another important objective of the present work was to test whether carnitine, which was recently shown to have anti-inflammatory and antioxidant properties, could avert the deleterious effects of these treatments.

## Material and methods

### Chemicals

All chemicals were of analytical grade and purchased from Sigma-Aldrich (St Louis, MO, USA) unless otherwise stated. Solutions were prepared on the day of the experiments and the pH was adjusted to 7.2–7.4. Lysine (Sigma-Aldrich #L5501), L-carnitine (Sigma-Aldrich #C0283), glutaric acid (Sigma-Aldrich #G3407) and quinolinic acid (Sigma-Aldrich #Q1375) were diluted in phosphate-buffered saline (PBS).

### Animals

Wild-type (WT) and *Gcdh*^*−/−*^ littermates, both of C129SvEv background, were generated from heterozygotes and maintained at *CREAL* Animal House of *the* Universidade Federal do Rio Grande do Sul (UFRGS), Porto Alegre, Brazil. The animals were maintained on a 12:12 h light/dark cycle (lights on 07:00–19:00 h) in air-conditioned constant temperature (22 ± 1 °C) colony room, with free access to water and a normal protein (20%) commercial pellet chow (SUPRA, Porto Alegre, Brazil).

#### Ethical approval and animal welfare

This study was performed in strict accordance with the Principles of Laboratory Animal Care, National Institute of Health of United States of America, NIH, publication no. 85–23, revised in 2011, the International Guiding Principles for Biomedical Research Involving Animals. All experimental procedures were also approved by the Ethics Committee on Animal Use of the Universidade Federal do Rio Grande do Sul (CEUA-UFRGS; protocol no. 46065). All efforts were made to minimize suffering, discomfort, stress, and the number of animals necessary to produce reliable scientific data.

### Lysine (Lys), L-carnitine (Carn), glutaric acid (GA) and quinolinic acid (QA) administration

Six-day-old (PND6) WT and *Gcdh*^*−/−*^ mice were used in the experiments for the determination of mRNA expression of the inflammatory biomarkers and the protein content of pro-inflammatory cytokines For the measurement of gene expression, mice were randomly separated, marked, weighed and divided into eight groups: WT mice: 1) PBS + PBS + PBS; 2) PBS + PBS + GA; 3) PBS + QA + GA; 4) Carn + QA + GA; *Gcdh*^*−/−*^ mice: 5) PBS + PBS + PBS; 6) PBS + PBS + GA; 7) PBS + QA + GA; 8) Carn + QA + GA.

**Fig. 1:**
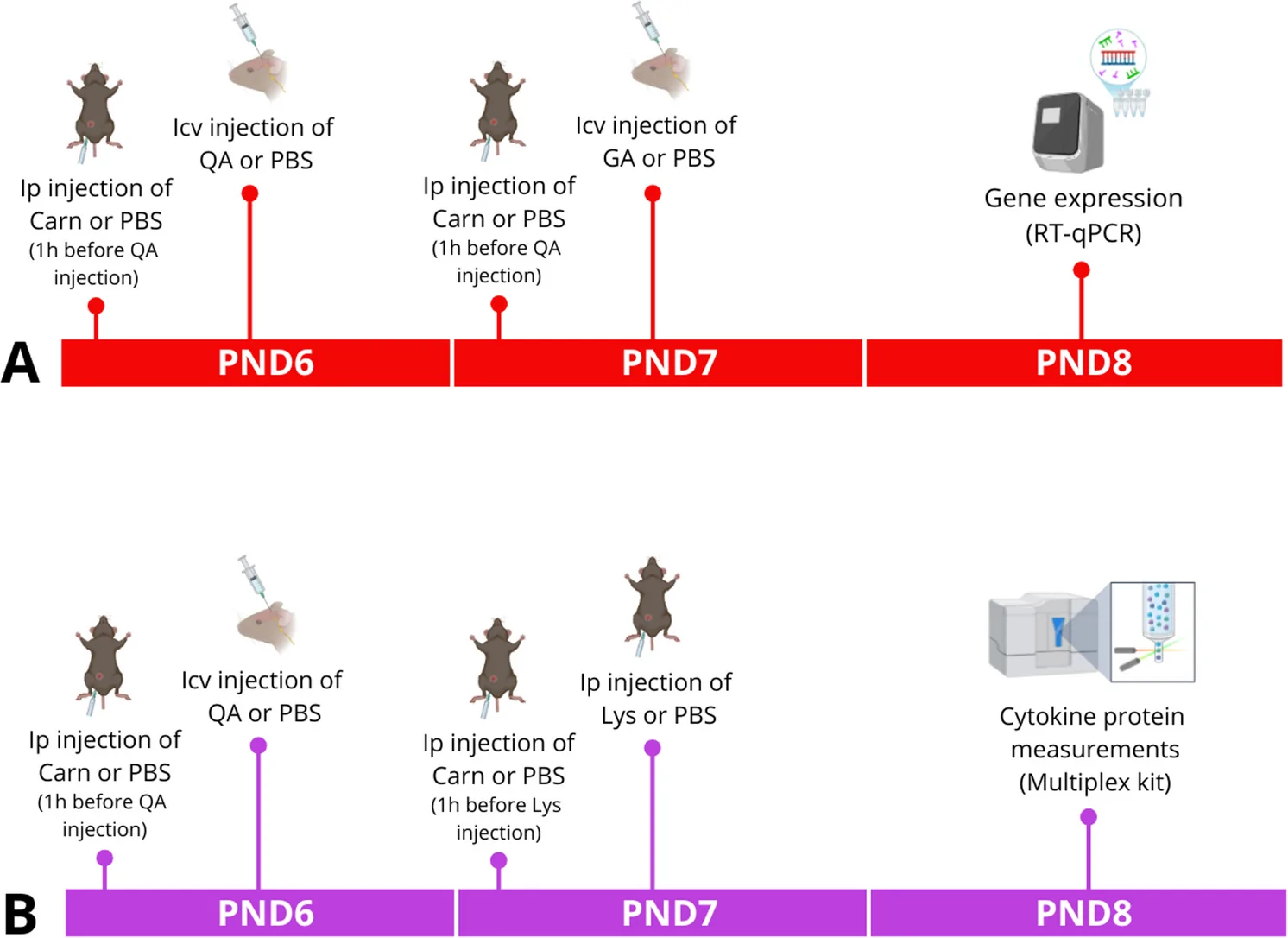
Schematic timelines of the experimental designs, showing animal treatments and mRNA (RT-qPCR) (Fig. 1 A) and protein (multiplex kit) (Fig. 1B) levels measured on postnatal day 8 (PND8). Male and female wild-type (WT) mice and GCDH gene knockout mice (*Gcdh*^*−/−*^) were used in the experiments. PND: postnatal day; GA: glutaric acid; QA: quinolinic acid; RT-qPCR: quantitative reverse transcription polymerase chain reaction

The animals of groups 4 and 8 received an intraperitoneal (ip) injection of Carn (100 mg/kg) one hour before intracerebroventricular (icv) administration of QA or GA, while mice of groups 1–3 and 5–7 received PBS (vehicle) instead. Mice of groups 3, 4, 7 and 8 received an icv injection of QA (5.6 nmol/g) on PND6, and the remaining groups received PBS. On PND7, the animals of groups 2–4 and 6–8 received an icv injection of GA (1 μmol/g), while the other groups received vehicle. On PND8, all animals were deeply anesthetized with isoflurane, euthanized and the cerebral cortex was dissected, identified, and prepared according to each technique. Figure 1A illustrates the injection protocol.

Furthermore, the protein content of pro-inflammatory cytokines was determined using an alternative protocol involving the ip administration of Lys (4 μmol/g), which is mainly converted to GA in the brain, instead of GA. For this study, WT and *Gcdh*^*−/−*^ were also divided into eight groups: 1) PBS + PBS + PBS; 2) PBS + Lys + PBS; 3) PBS + Lys + QA; 4) Carn + Lys + QA; *Gcdh*^*−/−*^ mice*:* 5) PBS + PBS + PBS; 6) PBS + Lys + PBS; 7) PBS + Lys + QA; 8) Carn + Lys + QA. Apart from ip Lys injection, the protocol was identical to that using GA administration (Fig. 1B).

### Quantitative RT-PCR

Quantitative RT-PCR was used to determine relative mRNA levels. Total RNA was extracted from cerebral cortex samples using TRI Reagent® (Sigma-Aldrich, #T9424, USA). RNA concentration and purity were assessed using a NanoDrop™ spectrophotometer (Thermo Fisher Scientific) based on absorbance at 260 nm and the A260/A280 ratio, respectively. Two micrograms of total RNA were reverse-transcribed using the High-Capacity cDNA Reverse Transcription Kit (Thermo Fisher Scientific, #4,368,814). Cerebral cortex samples from wild-type (WT) and *Gcdh*^*−/−*^ mice were collected at PND8, 24 h after the GA or vehicle injection. Relative transcript levels were determined for genes encoding proteins associated with inflammatory signaling: *PTGS2* (COX-2; Mm00478374_m1), *NOS2* (iNOS; Mm00440502_m1), *NFKB1* (NF-κB; Mm00476361_m1), *NFKBIA* (IκBα; Mm00477798_m1), *NLRP3* (NLRP3; Mm00840904_m1), and *TLR2* (TLR2; Mm00442346_m1); pro-inflammatory cytokines: *IL6* (IL-6; Mm00446190_m1), *IL1B* (IL-1β; Mm00434228_m1), and *TNF* (TNF-α; Mm00443258_m1); the anti-inflammatory cytokine *IL10* (IL-10; Mm01288386_m1); endoplasmic reticulum stress-related proteins: *DDIT3* (CHOP; Mm01135937_g1) and *EIF2AK3* (PERK; Mm00438700_m1); and neurotrophic and vascular factors: *VEGFA* (VEGF-A; Mm00437306_m1), *BDNF* (BDNF; Mm04230607_s1), and *NTRK2* (TrkB; Mm00435422_m1). *ACTB* (β-actin; Mm02619580_g1) was used as the reference gene for normalization. All TaqMan™ Gene Expression Assays were obtained from Thermo Fisher Scientific. Relative mRNA levels were calculated using the 2^ − ΔΔCt method (Livak and Schmittgen 2001).

### Cytokine determination

The protein content of the pro-inflammatory cytokines IL-1β, IL-6, and TNF-α was determined in cerebral cortex from WT and *Gcdh*^*−/−*^ mice euthanized on PND8, 24 h after injections of Lys and QA. Cytokine measurements were performed simultaneously using a ProcartaPlex™ Mouse Simplex panel (#PPX-MXKA6U3, Invitrogen), with assays conducted in duplicate according to the manufacturer’s protocol for sample dilution and concentration, in addition to a standard curve. Results are expressed in ρg/mL.

### Statistical analysis

The obtained data underwent a normality test using the Shapiro–Wilk method. The data are presented as mean ± standard deviation and analyzed by two-way ANOVA followed by the post hoc Tukey’s multiple range test when F was significant. Differences between groups were rated significant at *P* < 0.05. All analyses were carried out using GraphPad Prism software (version 10.1.2).

## Results

### L-carnitine modulates gene expression alterations of key neuroinflammatory signaling pathways induced in vivo by glutaric acid and quinolinic acid in cerebral cortex of GCDH deficient mice

**Fig. 2:**
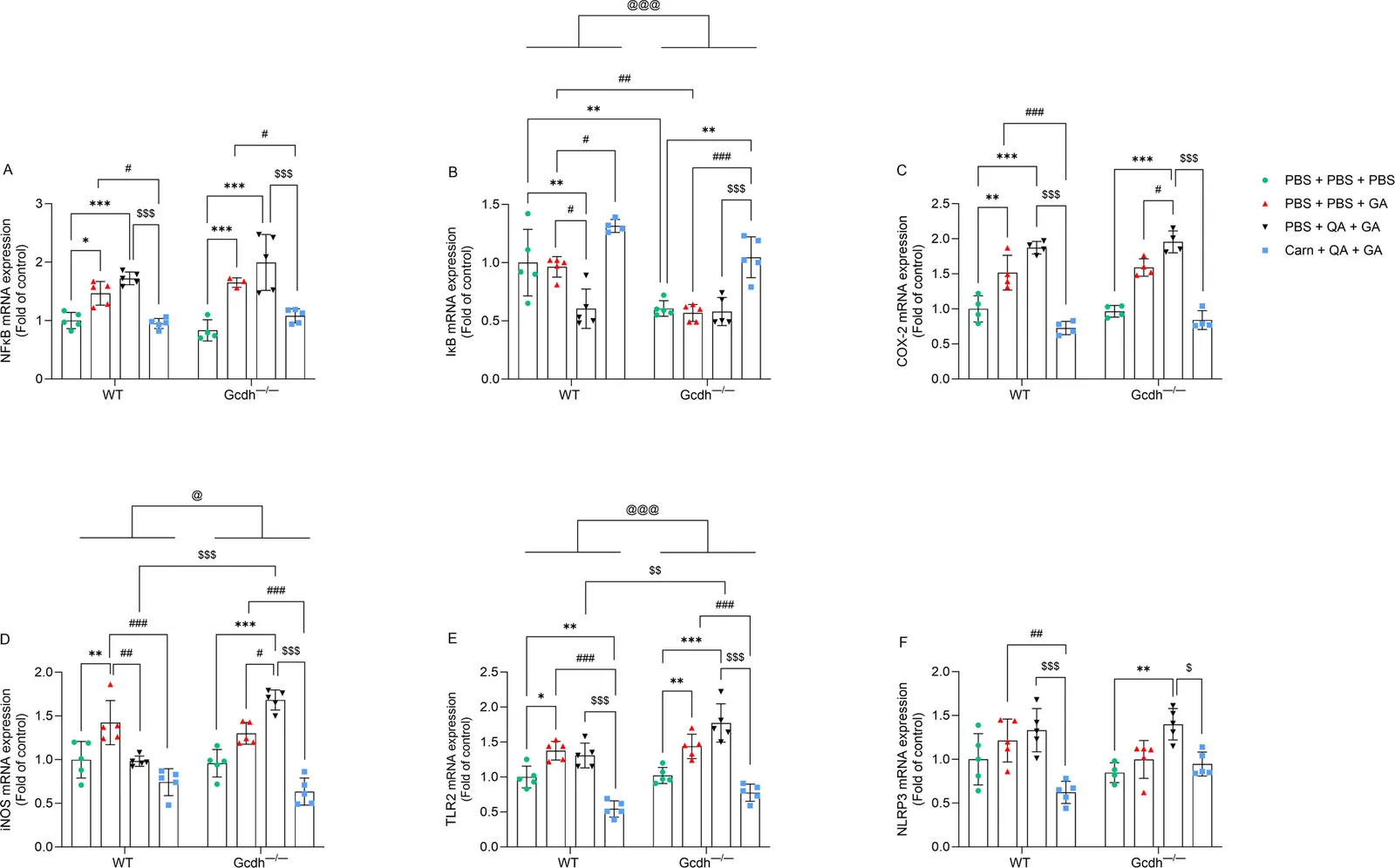
Alterations of mRNA gene expression of the neuroinflammatory signaling pathways NF-κB (**A**), IκBα (**B**), COX-2 (**C**), iNOS (**D**), TLR2 (**E**) and NLRP3 (**F**), caused by icv injections of QA and GA in the cerebral cortex of WT and *Gcdh*^*−/−*^ mice on PND8. Data are presented as mean ± standard deviation (SD), with n = 3–5 animals per group. Statistical analysis was performed using two-way ANOVA followed by the Tukey’s post hoc test (*P < 0.05, **P < 0.01, ***P < 0.001, differences from PBS + PBS + PBS; ^#^*P* < 0.05, ^##^*P* < 0.01, ^###^*P* < 0.001, differences from PBS + PBS + GA; ^$$$^*P* < 0.001 differences from PBS + QA + GA; ^@^*P* < 0.05, ^@@@^*P* < 0.001 differences from genotype)

Initially, we evaluated the effects of glutaric acid (GA), administered alone or in combination with quinolinic acid (QA), on key components of the inflammatory signaling in the cerebral cortex of wild-type (WT) and GCDH-deficient (*Gcdh*^*−/−*^) mice. GA administration significantly upregulated the mRNA expression of NF-κB (Fig. 2A) (Treatment *P* < 0.001; Interaction *P* = 0.2487; Genotype *P* = 0.055), COX-2 (Fig. 2C) (Treatment *P* < 0.0001; Interaction *P* = 0.771; Genotype *P* = 0.2759), iNOS (Fig. 2D) (Treatment *P* < 0.0001; Interaction *P* < 0.0001; Genotype *P* < 0.05), and TLR2 (Fig. 2E) (Treatment *P* < 0.0001; Interaction *P* < 0,05; Genotype *P* < 0.001) in both genotypes, while no significant changes were observed in IκBα (Fig. 2B) and NLRP3 (Fig. 2F) expression. Notably, co-administration of GA and QA potentiated the pro-inflammatory effects induced by GA alone, indicating that QA expanded selected components of the inflammatory response induced by GA alone. This potentiation was particularly evident in *Gcdh*^*−/−*^ animals, which exhibited an exacerbated increase in the expression of NF-κB (A), COX-2 (C), iNOS (D), TLR2 (E), and NLRP3 (F). In addition, genotype-dependent differences were observed, as *Gcdh*^*−/−*^ mice displayed baseline alterations in inflammatory markers, including IκBα (Fig. 2B), iNOS (Fig. 2D), and TLR2 (Fig. 2E). Of note, IκBα (Fig. 2B) expression was significantly reduced in *Gcdh*^*−/−*^ animals compared to WT, suggesting a predisposition to enhanced NF-κB pathway activation in the *Gcdh*^*−/−*^ animals. Importantly, Carn treatment exerted a robust anti-inflammatory effect in animals subjected to combined GA and QA administration, effectively reversing the observed molecular alterations and restoring all evaluated parameters to levels comparable to those of the control group. Carn treatment significantly increased IκBα (Fig. 2B) expression, supporting a potential mechanism involving negative regulation of NF-κB signaling.

### L-carnitine modulates the increased pro-inflammatory cytokines and the reduced IL-10 mRNA expression induced in vivo by glutaric acid and quinolinic acid in cerebral cortex of GCDH deficient mice

**Fig. 3:**
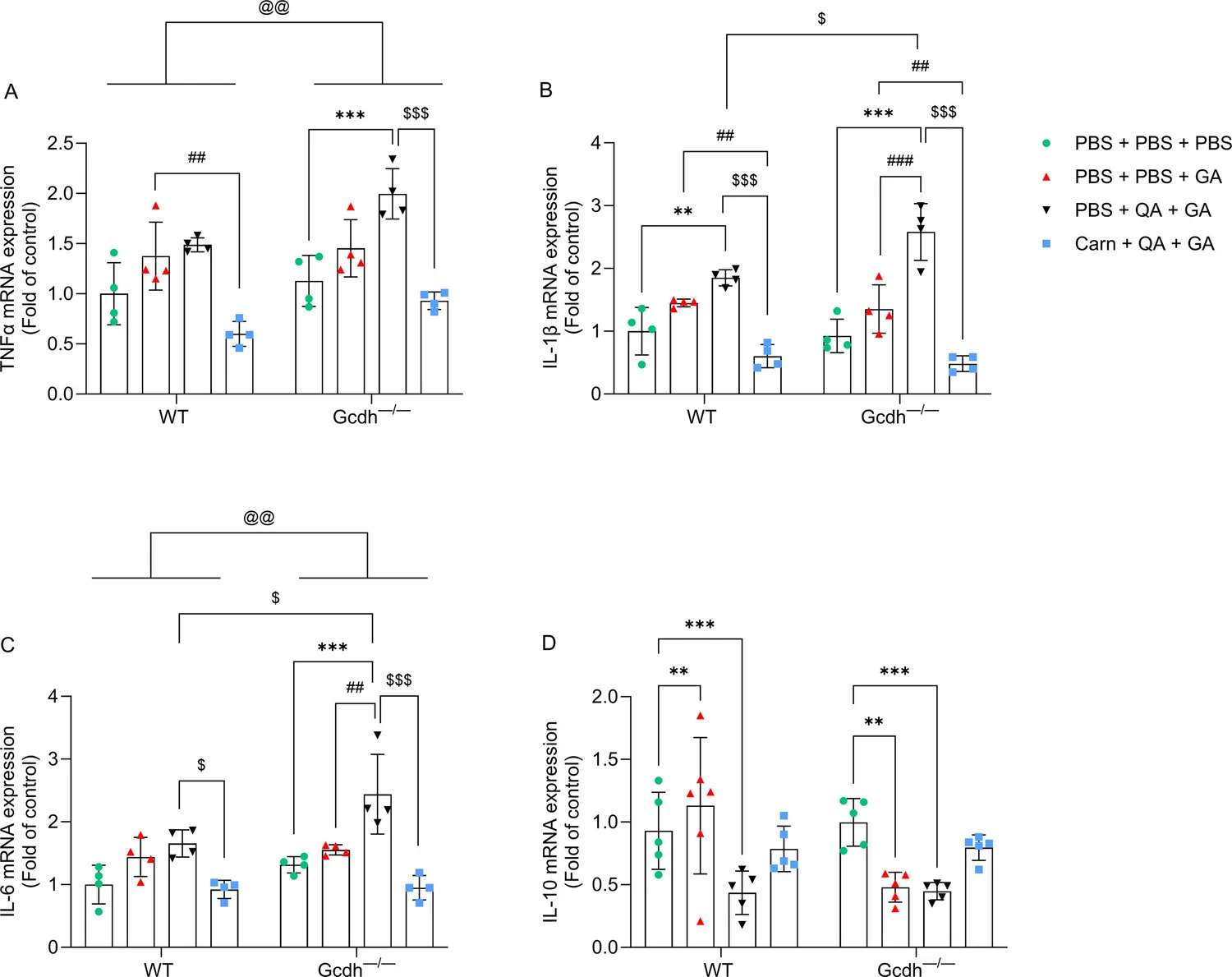
Changes of the mRNA expression of the pro-inflammatory cytokines TNF-α (**A**), IL-1β (**B**), and IL-6 (**C**) and the anti-inflammatory cytokine IL-10 (**D**) following QA and GA icv injections in the cerebral cortex of WT and *Gcdh*^*−/−*^ mice on PND 8. Data are presented as mean ± standard deviation (SD), with n = 3–5 animals per group. Statistical analysis was performed using two-way ANOVA followed by the Tukey’s post hoc test (**P < 0.01, ***P < 0.001, differences from PBS + PBS + PBS; ^##^*P* < 0.01, ^###^*P* < 0.001, differences from PBS + PBS + GA; ^$$^*P* < 0.01, ^$$$^*P* < 0.001 differences from PBS + QA + GA; ^@@^*P* < 0.01, differences from genotype)

Subsequently, we evaluated whether the alterations in inflammatory signaling pathways could modulate the gene expression of pro- and anti-inflammatory cytokines in the cerebral cortex. As shown in Fig. 3, administration of glutaric acid (GA) alone did not induce significant changes in the mRNA expression of TNF-α (3A) (Treatment *P* < 0.0001; Interaction *P* = 0.2680; Genotype *P* < 0.01), IL-1β (3B) (Treatment *P* < 0.0001; Interaction *P* < 0.05; Genotype *P* = 0.2842), or IL-6 (3C) (Treatment *P* < 0.0001; Interaction *P* = 0.0808; Genotype *P* < 0.01) in mice of both genotypes, but significantly reduced the mRNA levels of the anti-inflammatory cytokine IL-10 (3D) (Treatment *P* < 0.0001; Interaction *P* = 0.9557; Genotype *P* = 0.8545) in *Gcdh*^*−/−*^ animals. Furthermore, co-administration of GA and QA resulted in a significant increase in TNF-α (A), IL-1β (B), and IL-6 (C) expression in *Gcdh*^*−/−*^ mice, whereas in WT animals, a significant increase was observed only for IL-1β expression (B). Additionally, GA plus QA co-treatment led to a marked reduction in IL-10 (D) expression in both genotypes. Finally, and importantly, Carn exerted a protective effect by preventing the increase in the cytokines TNF-α (A), IL-1β (B), and IL-6 (C) in both WT and *Gcdh*^*−/−*^ mice, supporting a modulatory role of this compound in the inflammatory response triggered by these metabolites.

### L-carnitine normalizes the increased protein levels of pro-inflammatory cytokines caused in vivo by lysine and quinolinic acid in cerebral cortex of GCDH deficient mice

**Fig. 4:**
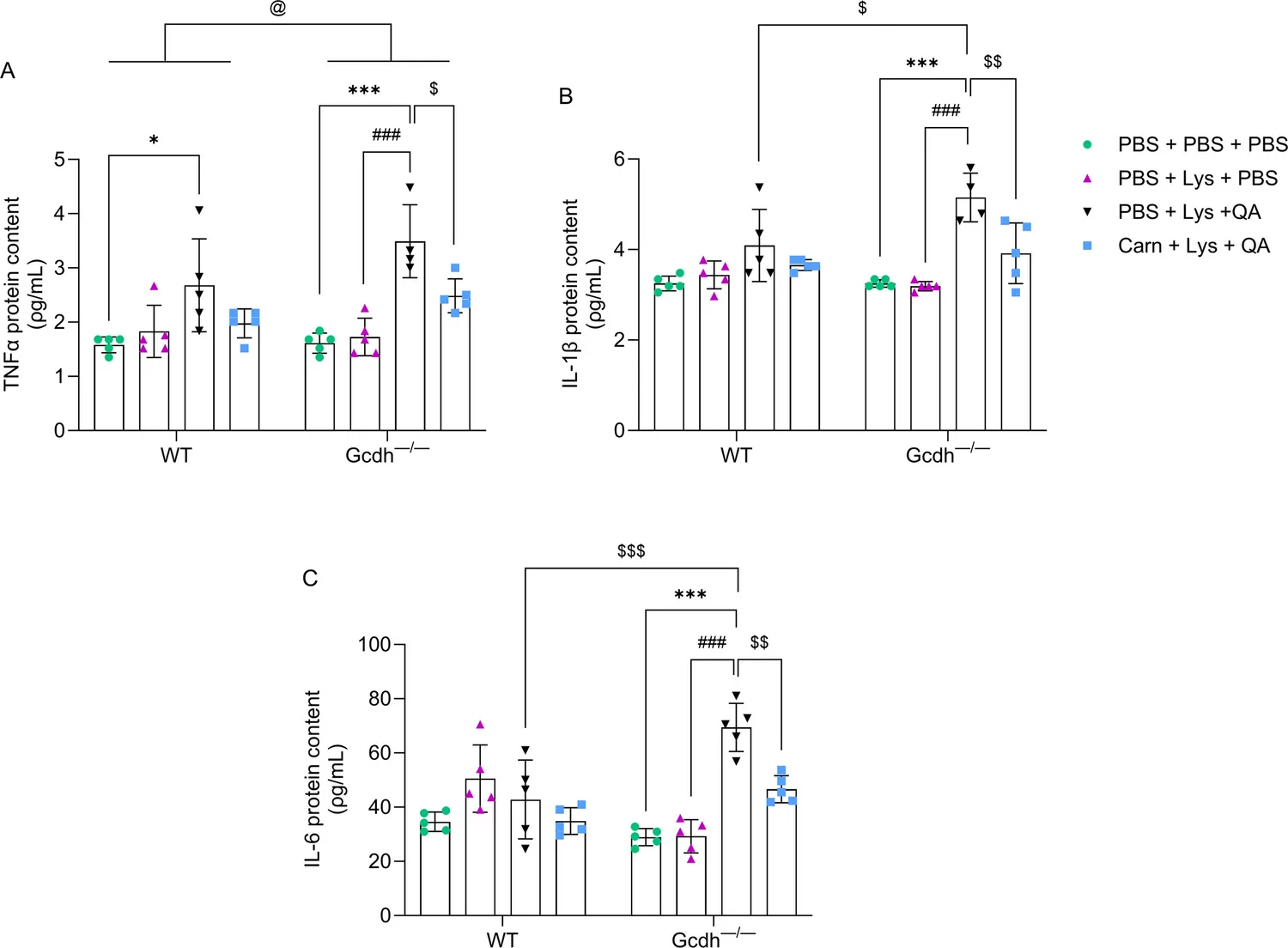
Changes of cortical protein levels of the pro-inflammatory cytokines TNF-α (**A**), IL-1β (**B**), and IL-6 (**C**) following QA (icv) and Lys (ip) injections in WT and *Gcdh*^*−/−*^ mice on PND 8. Data are presented as mean ± standard deviation (SD), with n = 4–5 animals per group. Statistical analysis was performed using two-way ANOVA followed by the Tukey’s post hoc test (*P < 0.05, ***P < 0.001 differences from PBS + PBS + PBS; ^###^*P* < 0.001, differences from PBS + Lys + PBS; ^$^*P* < 0.05; ^$$^*P* < 0.01, ^$$$^*P* < 0.001 differences from PBS + Lys + QA; ^@^*P* < 0.05 differences from genotype)

Next, we measured the protein levels of the pro-inflammatory cytokines TNF-α, IL-1β and IL-6 in the cerebral cortex of WT and *Gcdh*^*−/−*^ after acute co-administration of QA and lysine (Lys), which is metabolized to GA in the CNS of *Gcdh*^*−/−*^ animals. We found that Lys combined with QA treatment significantly enhanced the protein levels of the pro-inflammatory cytokines TNF-α (Treatment *P* < 0.0001; Interaction *P* = 0.1350; Genotype *P* < 0.05) (Fig. 4A), IL-1β (Treatment *P* < 0.0001; Interaction *P* < 0.05; Genotype *P* = 0.0626) (Fig. 4B), and IL-6 (Treatment *P* < 0.0001; Interaction *P* < 0.0001; Genotype *P* = 0.2843) (Fig. 4C). Furthermore, Lys administration alone did not alter the levels of any of the cytokines analyzed in both genotypes. In contrast and consistent with the mRNA expression data obtained in the GA plus QA model, the combined administration of Lys and QA resulted in a marked increase of all pro-inflammatory cytokines in the *Gcdh*^*−/−*^ animals. However, co-administration of QA and Lys caused a minimal effect in WT mice, with only a slight increase observed for TNF-α. Taken together, these findings indicate a genotype-dependent enhancement of the inflammatory response. Noteworthy, treatment with Carn significantly prevented the increase of all cytokines evaluated, highlighting its anti-inflammatory effect and protective role against the deleterious consequences of Lys and QA co-administration.

### L-carnitine mitigates endoplasmic reticulum stress induced in vivo by glutaric acid and quinolinic acid in cerebral cortex of GCDH deficient mice

**Fig. 5:**
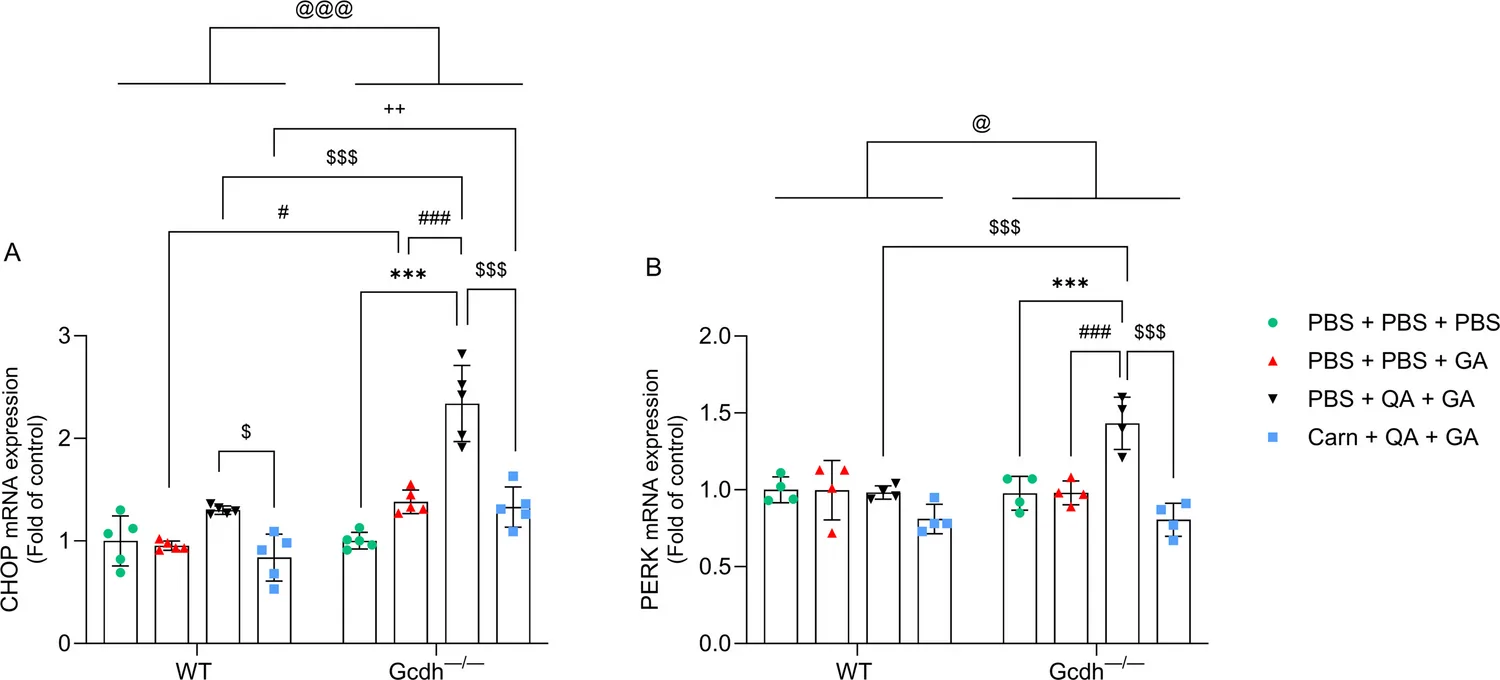
mRNA expression of CHOP (**A**) and PERK (**B**) following QA and GA icv injections in cerebral cortex of WT and *Gcdh*^*−/−*^ mice on PND8. Data are presented as mean ± standard deviation (SD), with n = 4–5 animals per group. Statistical analysis was performed using two-way ANOVA followed by the Tukey’s post hoc test (***P < 0.001, differences from PBS + PBS + PBS; ^#^*P* < 0.05, ^###^*P* < 0.001, differences from PBS + PBS + GA; ^$$$^*P* < 0.001 differences from PBS + QA + GA; ^++^*P* < 0.01 differences from Carn + QA + GA; ^@^*P* < 0.05, ^@@@^*P* < 0.001 differences from genotype)

Given that inflammatory signaling is a well-established trigger of endoplasmic reticulum (ER) stress, we next examined whether the neuroinflammatory alterations induced by GA and QA were associated with activation of ER stress pathways in the cerebral cortex. Co-administration of GA and QA markedly increased CHOP (Treatment *P* < 0.0001; Interaction *P* < 0.0001; Genotype *P* < 0.0001) (Fig. 5A) and PERK (Treatment *P* < 0.0001; Interaction *P* < 0.001; Genotype *P* < 0.05) (Fig. 5B) mRNA levels in *Gcdh*^*−/−*^ animals, indicating an activation of the ER stress response. Finally, treatment with Carn effectively prevented the GA + QA-induced upregulation of CHOP and PERK, restoring their expression to levels comparable to controls, suggesting a protective role of Carn against ER stress activation.

### L-carnitine restores the reductions in BDNF and VEGF mRNA expression induced in vivo by glutaric acid and quinolinic acid in cerebral cortex of GCDH deficient mice

**Fig. 6:**
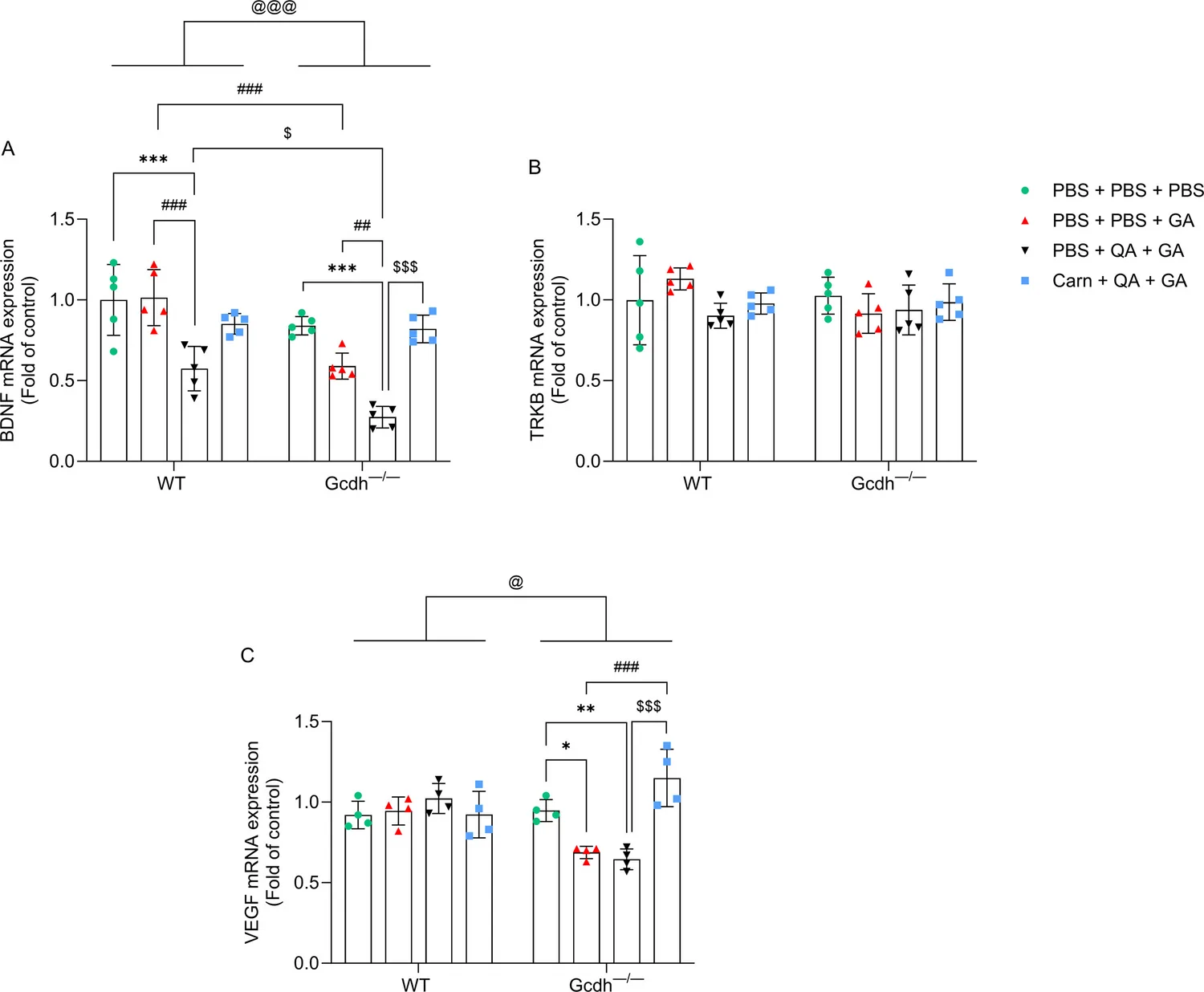
Alterations of mRNA of neurotrophic factors BDNF (**A**), VEGF (**C**), and TRKB (**B**) a receptor of BDNF following QA and GA icv injections in cerebral cortex of neonatal WT and *Gcdh*^*−/−*^ mice. Data are presented as mean ± standard deviation (SD), with n = 4–5 animals per group. Statistical analysis was performed using two-way ANOVA followed by the Tukey’s post hoc test (*P < 0.05, **P < 0.01, ***P < 0.001, differences from PBS + PBS + PBS; ^##^*P* < 0.01, ^###^*P* < 0.001, differences from PBS + PBS + GA; ^$^*P* < 0.05, ^$$$^*P* < 0.001 differences from PBS + QA + GA; ^@^*P* < 0.05, ^@@@^*P* < 0.001 differences from genotype)

We next assessed the effects of icv administration of GA and QA on the mRNA expression of the neurotrophic factors BDNF and VEGF, as well as on the BDNF receptor TRKB. As shown in Fig. 6, GA alone selectively reduced VEGF mRNA levels (Treatment *P* < 0.0001; Interaction *P* < 0.0001; Genotype *P* < 0.05) (Fig. 6C), without affecting BDNF (Treatment *P* < 0.0001; Interaction *P* < 0.001; Genotype *P* < 0.0001) (Fig. 6A) or TRKB (Treatment *P* = 0,3657; Interaction *P* = 0,1611; Genotype *P* = 0.4275) (Fig. 6B) expression in *Gcdh*^*−/−*^ mice. In contrast, co-administration of GA and QA induced a marked reduction in BDNF mRNA levels in both genotypes, with a more pronounced effect observed in *Gcdh*^*−/−*^ animals. Notably, VEGF expression was decreased only in *Gcdh*^*−/−*^ mice under these conditions, indicating a genotype-dependent vulnerability. TRKB mRNA levels remained unchanged across all experimental groups. Finally, treatment with Carn effectively reversed the alterations induced by GA plus QA co-administration, restoring both BDNF and VEGF expression to levels comparable to controls.

## Discussion

Systemic infections can worsen neurological manifestations of inborn errors of metabolism of intoxication, like the organic acidemias, by triggering encephalopathy that can be life-threatening. By causing fever and inflammation, infections accelerate protein breakdown inducing a catabolic state and acute metabolic decompensation resulting in accumulation of toxic metabolites that cause further biochemical perturbations, cellular and brain damage.

Regarding glutaric aciduria type I (GA-I), infections are the most common cause that trigger metabolic crises ultimately leading to irreversible striatal damage in children between 6 and 36 months of age, and this seems to be correlated to the predominant accumulation of glutaric acid and 3-hydroxyglutaric acid in the brain (Goodman et al. 1977a; Kölker et al. 2006; Wajner 2019). Aggressive management with prompt identification and treatment of infections is therefore critical to prevent severe decompensation and acute striatum degeneration in this disease (Goodman et al. 1977b; Kölker et al. 2006; Harting et al. 2009; Boy et al. 2013). Furthermore, rapid diagnosis through newborn screening and early treatment onset based on low protein/lysine diet allied to carnitine supplementation significantly ameliorates and reduces the severity of these crises, highligthing the importance of reducing the tissue levels of the accumulating metabolites (Kölker et al. 2011; Heringer et al. 2016; Strauss et al. 2020).

On the other hand, little is known about the influence of infectious episodes on the extrastriatal findings like the cortical abnormalities and atrophy in GA I. In this particular, frontotemporal hypoplasia and subependymal nodules are routinely found in MRI of late-onset GA I patients, who do not have an apparent susceptibility for striatal injury despite lack of treatment. These patients with no history of infections-associated crises are characteristically diagnosed over 6 years of age. Cerebral abnormalities at this age are well developed and thought to be due to sustained cumulative neurotoxicity of the accumulating metabolites, particularly GA (Boy et al. 2017, 2018; Wajner 2019). It is stressed that to the best of our knowledge practically nothing has been yet reported on the role of infectious processes on the brain alterations in these patients. Therefore, the present study was designed to investigate the role of an acute overload of GA under an increased inflammatory response triggered by icv injection of QA on neuroinflammatory signaling pathways, endoplasmic reticulum stress, as well as the levels of neurotrophins in cerebral cortex of neonatal *Gcdh*^*−/−*^ mice to clarify whether cortical lesions could be accelerated by infectious episodes. We also evaluated the beneficial effects of carnitine, which was recently reported to have potent anti-inflammatory and antioxidant properties, to test whether this compound could ameliorate the deleterious effects of GA and QA treatments.

Previous studies demonstrated that brain accumulation of GA promotes neuronal damage secondary to oxidative stress and excitotoxicity, culminating in glial activation (Olivera-Bravo et al. 2015; Amaral et al. 2018; Pierozan et al. 2018; Castro et al. 2024). However, the neurotoxic mechanisms triggered by the intracerebral accumulation of GA, especially those related to the inflammatory response, are still poorly understood.

In this work we investigated the influence of GA icv administration alone or combined with QA to WT and *Gcdh*^*−/−*^ mice on a range of important neuroinflammatory parameters. Our results showed that GA alone altered the expression of selected genes of inflammatory signaling pathways, increasing the mRNA levels of genes encoding NF-κB, COX-2, iNOS, and TLR2 in both genotypes. Combined QA + GA treatment produced a more intense molecular response, including increased TNF-α, IL-1β, IL-6, NLRP3, CHOP and PERK mRNA levels in the *Gcdh*^*−/−*^ mice, as well as reduced IκBα, IL-10, BDNF in both genotypes, and VEGF-A expression predominantly in the *Gcdh*^*−/−*^ mice. Therefore, the inflammatory transcriptional changes observed in this study cannot be attributed solely to the inflammatory stimulus mediated by QA. Overall, these marker-specific profiles indicate that QA selectively amplified downstream inflammatory responses to GA, particularly in the GCDH-deficient brain, rather than producing a uniform additive effect across all evaluated genes. TLR2 recognizes damage-associated molecular patterns and promotes NF-κB-dependent inflammatory signaling (Piccinini and Midwood 2010; Kawai and Akira 2010). The concomitant increases in TLR2 and IκBα expression after GA administration indicate early transcriptional engagement of this inflammatory network. In addition, *Gcdh*^*−/−*^ mice exhibited reduced basal IκBα expression, while QA + GA reduced IκBα expression in WT mice. Because IκBα negatively regulates NF-κB signaling, this expression profile is compatible with reduced transcriptional restraint of the inflammatory response. Increased expression of COX-2 and iNOS, which encode important downstream inflammatory enzymes, further supports the induction of a coordinated pro-inflammatory molecular program. The increase in NLRP3 observed after QA + GA exposure, predominantly in *Gcdh*^*−/−*^ mice, also indicates transcriptional priming of the NLRP3 inflammasome and suggests that GCDH deficiency increases susceptibility to the combined challenge (Swanson et al. 2019).

The cytokine profile further demonstrates that the combined QA + GA exposure expanded the response induced by GA. GA alone did not significantly alter TNF*-*⍺*,* IL-1β, or IL-6 expression but reduced the anti-inflammatory cytokine IL-10 in *Gcdh*^*−/−*^ mice. In contrast, QA + GA treatment increased TNF*-*⍺*,* IL-1β, or IL-6 expression in *Gcdh*^*−/−*^ mice, whereas IL-1β was also increased in WT animals. The maintenance of reduced IL-10 expression in the mutant animals, together with the induction of pro-inflammatory cytokines, indicates an imbalance between pro- and anti-inflammatory transcriptional responses. The more extensive response in *Gcdh*^*−/−*^ mice highlights the vulnerability imposed by GCDH deficiency and suggests that the accumulation of GA sensitizes the cerebral cortex to additional inflammatory-associated stimuli.

Complementary protein-level evidence was obtained using the Lys + QA experimental approach. Lys alone did not significantly alter cortical TNF-α, IL-1β, or IL-6 concentrations, whereas Lys + QA increased all three cytokines, predominantly in *Gcdh*^*−/−*^ mice. Because lysine is a major precursor of GA formation, these results provide complementary support for the interaction between GA brain increased levels and an inflammatory-associated challenge. Therefore, the present cytokine protein findings are in agreement with those of mRNA levels, implying that the results on the present investigation on gene expression are probably translated into protein levels.

Another novel finding of the present work was the observation that Carn attenuated or normalized most of the molecular alterations induced by the combined administration of QA and GA. In this protocol, Carn modulated the mRNA expression of key inflammatory mediators, including TLR2, NF-κB, IκBα, COX-2, iNOS, and NLRP3, as well as the pro-inflammatory cytokine genes TNF-α, IL-1β, and IL-6. Consistent with these transcriptional effects, Carn also attenuated or normalized the protein levels of TNF-α, IL-1β, and IL-6 in the complementary Lys + QA protocol. The consistency of its effects across inflammatory signaling genes, cytokine transcripts, and cytokine proteins strongly supports a broad modulatory action of Carn on the inflammatory response. These findings agree with recent studies demonstrating antioxidant and anti-inflammatory effects of Carn and its derivatives in experimental models of neurotoxicity and neuroinflammation (Zanelli et al. 2005; Ferreira and McKenna 2017; Kazak and Yarim 2017; Bigio et al. 2024). In the context of GA I, the present results suggest that the benefits of Carn may extend beyond the formation and elimination of glutarylcarnitine to include modulation of molecular responses elicited by inflammatory stress.

Combined QA + GA exposure also increased CHOP and PERK mRNA expression in *Gcdh*^−/−^ mice, indicating induction of an ER stress-related transcriptional response. PERK is a major sensor of disturbed ER homeostasis, whereas CHOP is associated with maladaptive responses to persistent ER stress (Hetz and Saxena 2017; Hetz et al. 2020). The parallel induction of inflammatory and ER stress-related genes is consistent with the close interaction between these processes, since inflammatory and metabolic stressors associated with redox imbalance, can disrupt ER proteostasis and activate the unfolded protein response (Hotamisligil 2010). Carn prevented or attenuated the increases in CHOP and PERK, suggesting that its modulatory effects also encompass molecular responses associated with ER homeostasis.

The inflammatory and ER stress-related disturbances were accompanied by altered expression of genes associated with neurotrophic and vascular support. In this scenario, QA + GA treatment reduced BDNF expression in both genotypes, with a greater effect in *Gcdh*^*−/−*^ mice, while VEGF-A was reduced only in the knockout animals and TRKB remained unchanged. BDNF is an important regulator of neuronal survival and synaptic plasticity, whereas VEGF-A exerts both vascular and neurotrophic actions. Pro-inflammatory cytokines can interfere with transcriptional pathways regulating these factors, providing a biologically plausible connection between the inflammatory response and their reduced expression (Barrientos et al. 2004; Patterson 2015; Zhang et al. 2016). Carn also modulated the reductions in BDNF and VEGF-A, indicating preservation of molecular components associated with neurotrophic and vascular support. These findings agree with the evidence that Carn derivatives can preserve BDNF-related responses and neuroplasticity-associated pathways in other experimental conditions (Kazak and Yarim 2017).

To the best of our knowledge, this is the first in vivo study to investigate the molecular responses elicited by GA alone and by the combined administration of GA and QA in the cerebral cortex of neonatal *Gcdh*^*−/−*^ mice, while concurrently examining innate immune signaling involving TLR2, NF-κB, and NLRP3, endoplasmic reticulum stress-related responses, neurotrophic and angiogenic support involving BDNF and VEGF-A and finally the modulatory neuroprotective effects of L-carnitine. This integrated approach provides a comprehensive overview of the molecular responses that may occur during infection-triggered inflammatory and metabolic stress in GA I.

The present study provides new evidence that GA alone induces an inflammatory-associated transcriptional response in the cerebral cortex, that QA expands selected components of this response, particularly in *Gcdh*^*−/−*^ mice, and that Carn attenuates molecular alterations associated with inflammation, ER stress, and neurotrophic support. These findings should nevertheless be interpreted within the scope of the experimental design. The acute intracerebroventricular approach enabled a controlled assessment of the direct cerebral responses to GA and QA, although it does not fully reproduce the gradual endogenous accumulation of metabolites or the systemic inflammatory and catabolic components of metabolic decompensation in patients with GA I. Similarly, the absence of a QA-only group limits the assessment of its independent effects and prevents a formal determination of synergism, but the comparison between GA and QA + GA allowed the identification of molecular responses selectively potentiated by the combined challenge. The transcriptional analysis provided a comprehensive and biologically coherent profile of the pathways affected, complemented by cytokine protein measurements in the protocol using Lys + QA treatment. Further studies incorporating additional protein and pathway-activation analyses, histological and behavioral outcomes, glial responses, and mitochondrial function will help establishing the cellular and functional consequences of these molecular alterations and clarify the mechanisms underlying Carn protection. Evaluation of additional Carn doses and treatment periods will also be valuable to extend the present proof-of-concept findings.

Taken together, our findings demonstrate that GA alone induces the expression of some inflammatory-associated genes and that QA + GA combined treatment expands this response, producing more extensive alterations in biomarkers of inflammatory and related ER stress, besides altering neurotrophic molecular pathways, particularly in *Gcdh*^*−/−*^ mice. Carn markedly attenuated these transcriptional changes and reduced cytokine protein elevations in the complementary Lys + QA protocol. These results support the involvement of inflammatory-associated molecular mechanisms in cortical vulnerability in GA I and identify Carn as an important modulator of the cerebral response to combined metabolic and inflammatory challenges.

The pathophysiologic relevance of the data here disclosed are still uncertain. However, in case our present findings can be extrapolated to the human condition, it is conceivable that acute episodes of encephalopathy triggered by infections may be associated with the cortical abnormalities and atrophy observed by cerebral MRI in GA I patients. Thus, preventing these metabolic crises and a prompt treatment based on more aggressive measures during acute episodes of decompensation including higher doses of Carn may ameliorate the cortical alterations occurring in this disease.

We should emphasize that one limitation of this study was that we did not measure glutaric acid concentrations in the brain of animals injected with this organic acid. Instead, we based the dose administered to the animals on previous studies of our and other labs (Lima et al. 1998; de Mello et al. 2001; Külkens et al. 2005; Castro et al. 2024). Another limitation of the present work was that the translated protein levels were only measured for pro-inflammatory cytokines.

**Fig. 7:**
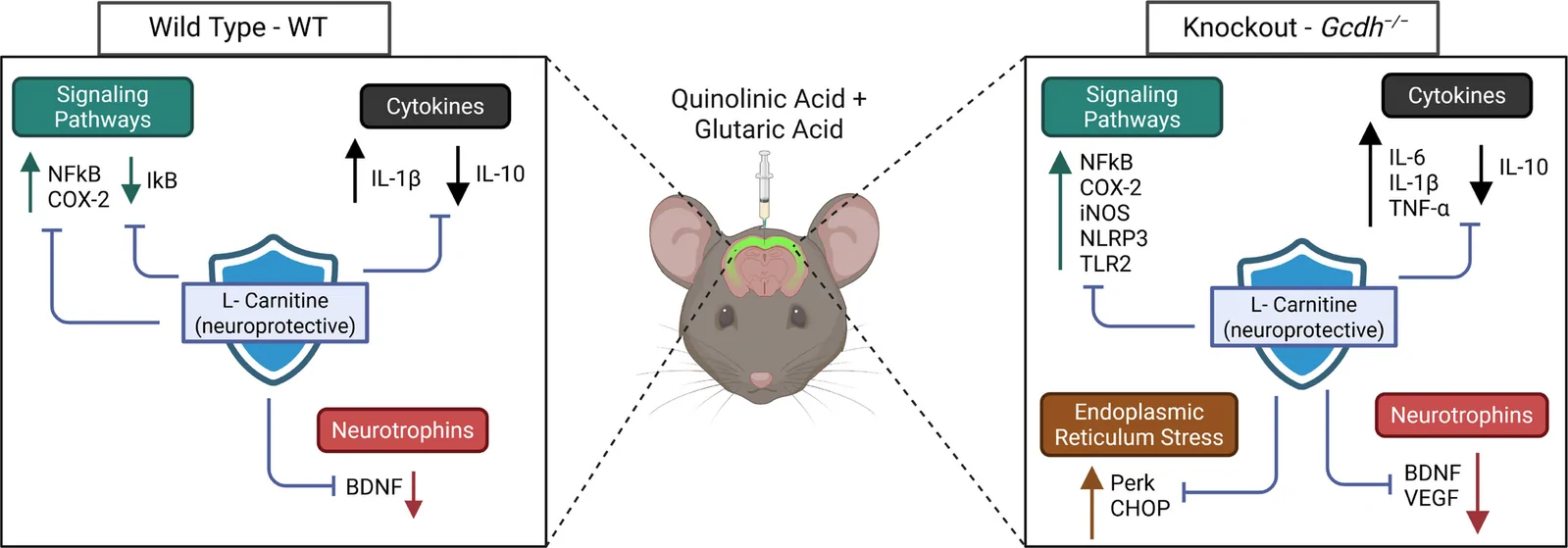
Schematic summary of the statistically significant molecular alterations observed following combined quinolinic acid (QA) and glutaric acid (GA) treatment in the cerebral cortex of wild-type (WT) and GCDH-deficient (Gcdh −/−) mice. Upward and downward arrows indicate increased and decreased expression, respectively. The diagram also illustrates the modulatory effects of L-carnitine, which attenuated or normalized most of the alterations associated with inflammatory signaling, cytokine expression, endoplasmic reticulum stress, and neurotrophic support

Figure 7 depicts a schematic representation of the deleterious effects of GA alone and GA + QA treatments on inflammatory response and signaling pathways, ER stress and neurotrophins levels, as potential pathomechanism involved in cerebral cortex damage in *Gcdh*^*−/−*^ mice. The figure also shows the neuroprotective role of L-carnitine.

## Supplementary Information

Supplementary Material 1

## Data Availability

All data supporting the results of this study are available in the article, and any additional data will be made available upon request.
