# Supplementary material for: L-carnitine modulates molecular responses associated with inflammation, endoplasmic reticulum stress, and neurotrophic signaling in cerebral cortex of neonatal Gcdh−/− mice exposed to glutaric and quinolinic acids
Source: Metab Brain Dis. 2026 Sep 5;41(1):205. doi: 10.1007/s11011-026-01973-y (PMC13546310; doi:10.1007/s11011-026-01973-y)
Supplement: Supplementary file 1 [file 11011_2026_1973_MOESM1_ESM.docx]

**Supplementary Material**

**Table S1.** Two-way ANOVA analyses for gene expression and cytokine protein levels in the cerebral cortex.

| **Fig.** | **Endpoint** | **Treatment** | **Genotype** | **Treatment × genotype** |
| --- | --- | --- | --- | --- |
| **2A** | *Nfkb1* mRNA | F(3,32) = 4.646 ***P*<0.01** | F(1,32) = 3.949 *P* = 0.0550 | F(3,32) = 1.442 *P* = 0.2487 |
| **2B** | *Nfkbia* mRNA | F (3,31) = 25,12  ***P*<0.0001** | F (1,31) = 31,41  ***P*<0.0001** | F (3,31) = 3,379  *P = 0,0306* |
| **2C** | *Ptgs2* mRNA | F(3,24) = 96.95 ***P*<0.0001** | F(1,24) = 1.243 *P* = 0.2759 | F(3,24) = 0.3754 *P* = 0.7710 |
| **2D** | *Nos2* mRNA | F(3,32) = 38.53 ***P*<0.0001** | F(1,32) = 4.315 ***P*<0.05** | F(3,32) = 14.85 ***P*<0.0001** |
| **2E** | *Tlr2* mRNA | F(3,32) = 57.66 ***P*<0.0001** | F(1,32) = 13.78 ***P*<0.001** | F(3,32) = 3.664 ***P*<0.05** |
| **2F** | *Nlrp3* mRNA | F (3,31) = 14,72  ***P<0,0001*** | F (1,31) = 0,007867  *P=0,9299* | F (3,31) = 3,457  *P=0,0282* |
| **3A** | *Tnf* mRNA | F(3,24) = 25.69 ***P*<0.0001** | F(1,24) = 9.730 ***P*<0.01** | F(3,24) = 1.397 *P* = 0.2680 |
| **3B** | *Il1b* mRNA | F(3,24) = 51.29 ***P*<0.0001** | F(1,24) = 1.200 *P* = 0.2842 | F(3,24) = 4.320 ***P*<0.05** |
| **3C** | *Il6* mRNA | F(3,24) = 20.66 ***P*<0.0001** | F(1,24) = 8.485 ***P*<0.01** | F(3,24) = 2.535 *P* = 0.0808 |
| **3D** | *Il10* mRNA | F(3,33) = 21.00 ***P*<0.0001** | F(1,33) = 0.03418 *P* = 0.8545 | F(3,33) = 0.1065 *P* = 0.9557 |
| **4A** | TNF-α protein | F(3,31) = 19.24 ***P*<0.0001** | F(1,31) = 4.459 ***P*<0.05** | F(3,31) = 1.996 *P* = 0.1350 |
| **4B** | IL-1β protein | F(3,31) = 19.66 ***P*<0.0001** | F(1,31) = 3.730 *P* = 0.0626 | F(3,31) = 3.951 ***P*<0.05** |
| **4C** | IL-6 protein | F(3,32) = 14.88 ***P*<0.0001** | F(1,32) = 1.186 *P* = 0.2843 | F(3,32) = 15.62 ***P*<0.0001** |
| **5A** | *Ddit3* mRNA | F(3,32) = 35.97 ***P*<0.0001** | F(1,32) = 61.83 ***P*<0.0001** | F(3,32) = 11.66 ***P*<0.0001** |
| **5B** | *Eif2ak3* mRNA | F(3,24) = 15.01 ***P*<0.0001** | F(1,24) = 5.745 ***P*<0.05** | F(3,24) = 7.581 ***P*<0.001** |
| **6A** | *Bdnf* mRNA | F(3,32) = 31.46 ***P*<0.0001** | F(1,32) = 34.09 ***P*<0.0001** | F(3,32) = 4.693 ***P*<0.01** |
| **6B** | *Ntrk2* mRNA | F(3,32) = 1.094 *P* = 0.3657 | F(1,32) = 0.6460 *P* = 0.4275 | F(3,32) = 1.832 *P* = 0.1611 |
| **6C** | *Vegfa* mRNA | F(3,24) = 7.641 ***P*<0.001** | F(1,24) = 6.672 ***P*<0.05** | F(3,24) = 13.88 ***P*<0.0001** |

**Note.** Values are reported as F values and probabilities. Statistically significant effects (P < 0.05) are shown in bold. For Figures 2, 3, 5, and 6, the treatment factor comprised the control, GA, QA+GA, and QA+GA+Carn groups; for Figure 4, it comprised the control, Lys, Lys+QA, and Lys+QA+Carn groups.
